# An Analysis of Variability in “CatWalk” Locomotor Measurements to Aid Experimental Design and Interpretation

**DOI:** 10.1523/ENEURO.0092-20.2020

**Published:** 2020-08-20

**Authors:** Miriam Aceves, Valerie A. Dietz, Jennifer N. Dulin, Unity Jeffery, Nicholas D. Jeffery

**Affiliations:** 1 Department of Small Animal Clinical Sciences, Texas A&M University, College Station, TX 77843; 2 Department of Biology, Texas A&M University, College Station, TX 77843; 3 Texas A&M Institute for Neuroscience, Texas A&M University, College Station, TX 77843; 4 Department of Veterinary Pathobiology, College of Veterinary Medicine, College Station, TX 77843

**Keywords:** outcome measure, spinal cord injury, translation

## Abstract

Preclinical studies in models of neurologic injury and disease rely on behavioral outcomes to measure intervention efficacy. For spinal cord injury, the CatWalk system provides unbiased quantitative assessment of subtle aspects of locomotor function in rodents and so can powerfully detect significant differences between experimental and control groups. Although clearly of key importance, summary group-level data can obscure the variability within and between individual subjects and therefore make it difficult to understand the magnitude of effect in individual animals and the proportion of a group that may show benefit. Here, we calculate reference change intervals (RCIs) that define boundaries of normal variability for measures of rat locomotion on the CatWalk. Our results indicate that many commonly-used outcome measures are highly variable, such that differences of up to 70% from baseline value must be considered normal variation. Many CatWalk outcome variables are also highly correlated and dependent on run speed. Application of calculated RCIs to open access data (https://scicrunch.org/odc-sci) on hindlimb stride length in spinal cord-injured rats illustrates the complementarity between group-level (16 mm change; *p* = 0.0009) and individual-level (5/32 animals show change outside RCI boundaries) analysis between week 3 and week 6 after injury. We also conclude that interdependence among CatWalk variables implies that test “batteries” require careful composition to ensure that different aspects of defective gait are analyzed. Calculation of RCIs aids in experimental design by quantifying variability and enriches overall data analysis by providing details of change at an individual level that complement group-level analysis.

## Significance Statement

Selection of robust candidate interventions for translation from experimental animals into the neurology clinic requires meticulous examination of behavioral effects observed in the laboratory. Although analysis of group-level data, the current mainstay, is critically important, analysis of individual-level data provides a complementary viewpoint that, bearing in mind the immense variability in neurologic deficits in people with spinal cord injury, has high relevance to the interpretation of studies on putative therapies. Here, we describe the derivation of specific reference change intervals (RCIs) and, using example data, show how these augment interpretation of overall effect and can aid in effective experimental design. The combination of group-level and individual-level analysis will provide more stringent analysis of intervention effects in neurologic injury and disease research.

## Introduction

Spinal cord injury research has two broad goals: to understand mechanisms by which injury causes tissue and functional loss and to develop methods of treatment that can be translated into the clinic. While the past three decades have seen substantial progress in achieving the first goal (Alizadeh et al., 2019), the second remains largely unfulfilled (Garner, 2014; Siddiqui et al., 2015; Eckert and Martin, 2017).

Depending on the functional target, there are many ways to define a successful experimental therapy, but, especially in view of the high costs, it is essential to identify truly effective interventions to carry forward to clinical trials. Standard analysis of outcome after an intervention designed to ameliorate the functional deficits caused by spinal cord injury relies on comparisons between groups of experimental animals and defines the population-level effect of an intervention. In contrast, the questions asked by a patient in the clinic are: “How likely am I, as an individual, to get benefit from this intervention?” and “How much benefit will I get?” Neither of these questions can be answered by group-level analysis, nor are benefits at an individual level guaranteed by detection of group-level efficacy (Rousselet et al., 2016).

Individual-level analysis has many complementary benefits. Importantly, it can reveal intraindividual and interindividual variability and thereby differentiate an intervention that produces an apparent difference between groups that is dependent on a large change in a small number of individuals from one that produces more widespread benefit throughout the group (Weissgerber et al., 2015; Rousselet et al., 2016). In addition, it can aid in quantifying benefits by putting the magnitude of the intervention effect into context through comparison with changes in outcome that can arise through spontaneous variability alone. This is most important at an individual level: spinal cord-injured people seek an intervention that will have substantial impact on their everyday lives and, to do so, such an intervention must have an effect that is greater than might arise through day-to-day variability alone. Interventions that produce reproducible benefits at both group and individual level can then be unequivocally recognized as appropriate candidates for translation.

Assessment of function following experimental spinal cord injury in animals has traditionally relied on observations of gait (Tarlov and Klinger, 1954), and nowadays most frequently through the BBB scale (Basso et al., 1995). Concerns about the nature of the BBB scale and its sensitivity in detecting non-stereotypical patterns of locomotor recovery, both of which could affect the reproducibility of outcomes (Steward et al., 2012), spurred the development of the CatWalk apparatus (Hamers et al., 2001; Koopmans et al., 2005). Its main advantage is that, through computerized analysis of locomotion on a walkway, it provides unbiased, quantitative data on multiple components of gait and paw placement. CatWalk analysis is now widely used to objectively quantify outcomes in spinal cord-injured rodents and control and intervention groups can be compared to assess efficacy of proposed novel therapeutics. To date, it been used to detect differences between groups of animals, but, in line with the objectives outlined above, it also provides data that are amenable to analysis of individual responses.

All measurement methods are susceptible to variability, which arises from factors both within and external to each individual. A key component of individual-level analysis is partitioning sources of variability; appropriate methods have been developed in hospital clinical laboratories so that an individual’s disease progress or response to therapy can be monitored. Sources of variability must be analyzed in individuals at a plateau of health or disease and can be appropriately allocated through repeated measures on small numbers (approximately eight or more) of normal individuals (Fraser and Harris, 1989; Braga and Panteghini, 2016). In this study, we used the same approach to define expected boundaries for individual variability of behavioral function on the CatWalk. We also aimed to define clearly the exact methods that were used for obtaining the data, with a view to simplifying comparison of data between and within laboratories, thereby enhancing reliability and reproducibility. Because CatWalk produces a large range of outcomes, we initially used PubMed to survey recent publications to identify frequently reported outcomes after spinal cord injury. The variability in these commonly-used outcomes was then quantified in a group of young adult rats by making repeat measures of their function over an eight-week period. Finally, we examined correlation among outcome measures to identify combinations of measures that are most likely to provide independent outcome data.

## Materials and Methods

All animal procedures were performed in accordance with the Texas A&M University institutional animal care and use committee’s regulations.

### Subjects

The subjects were male Sprague Dawley rats (*N* = 16) obtained from Envigo. Upon arrival they were approximately nine weeks old (250–275 g) and were pair-housed in standard Plexiglas cages with a 12/12 h light/dark cycle (changing at 7 A.M. and 7 P.M.) and food and water provided *ad libitum*. Subjects remained uninjured for the duration of the experiment, which consisted of a 5-d training period before weekly testing over a total period of eight weeks.

### CatWalk settings

We used CatWalk XT version 10.6 (Noldus) for this study. The glass walkway was adjusted so that it was slightly >8 cm wide and the camera was positioned 75 cm below it, allowing the virtual walkway size to be set at 70 cm long by 8 cm wide. Before beginning the experiment, camera detection settings were adjusted using the Auto Detect function in the program. The system was calibrated each time the camera position was adjusted using a 20 × 10 cm rectangular calibration sheet. Table 1 shows the values used throughout the experiment.

**Table 1:** CatWalk detection settings

Camera detection settings	Results	Auto detection settings
Camera gain (dB): 12.00 Green intensity threshold: 0.14 Red ceiling light (V): 17.70 Green walkway light (V): 16.0	Maximum green intensity: 0 Minimum green intensity: 256 Range: –256	Maximum range from 197 to 203 Frames before Delta: 5 Intensity minimum: 85

### Behavioral testing

First, to facilitate training and testing on the CatWalk, subjects were acclimated to a food reward (FrootLoops) placed in the home cage for three consecutive days, with no other activity. Training commenced immediately after food acclimation and for a total of 5 d. All training and testing sessions were conducted by the same researcher (M.A.) in a dark room at a consistent time of day (beginning at 9 A.M.). Before each session, animals were habituated to the testing room for 30 min.

On the first day of training, the rats were introduced to the testing environment and CatWalk apparatus. First, they were moved to the testing room in their home cages and left undisturbed for 30 min. Then they were placed on the CatWalk individually and allowed to explore freely for a period of 10 min. Care was taken to ensure that the walkway was cleaned thoroughly before and after each subject. At the end of the session, the rats were returned in their home cages to the vivarium. On each of the following 4 d, the rats were trained to cross the CatWalk: following a 30-min acclimation to the room, they were placed at one end of the walkway and encouraged to walk across to the other end for a food reward. The training session was terminated once the animal successfully completed three full runs across the walkway or reached a maximum time of 10 min on the CatWalk.

Baseline test data were acquired on the day immediately following the training period and then once weekly for the next seven weeks. During each testing session, subjects were required to complete three compliant runs, which, for this study, were defined by continuous, uninterrupted locomotion that traversed the entire walkway in either direction. Further criteria were also specified using the CatWalk program, as described in Table 2.

**Table 2:** Limits used to define a compliant run

Run criteria
Minimum run duration: 0.5 s Maximum run duration: 5.00 s Minimum number of compliant runs to acquire: 3 Use maximum allowed speed variation (left unchecked)

### Selection of popular CatWalk outcome measures

A previous publication (Kappos et al., 2017) identified four variables as being most commonly used in CatWalk analysis (albeit for analysis of hindlimb nerve function): swing duration, (paw) print size, stride length, and maximum (paw) contact area. In this study, we conducted a similar search in PubMed but limited the search to only include studies on spinal cord injury in rats; our search terms were: “rat,” “spinal cord injury,” “Catwalk.” The search hits were then examined to extract the most commonly analyzed outcomes.

### Analysis of example data

As an illustration of the value that can be added by using this new method we analyzed open source material available at odc-sci.org (https://scicrunch.org/odc-sci/lab/view-dataset?labid=51&datasetid=26). These data were collected as part of an experiment to examine the relationships between different behavioral outcome measures following spinal cord injury (Ferguson et al., 2013) and the raw data made publicly available. Our analysis here is simply to demonstrate how the method can be applied to an experimental dataset that is available for readers to investigate for themselves and not to provide alternative interpretations of the data. The rats in that experiment were trained to cross the CatWalk before induction of a cervical spinal cord injury using the MASCIS/NYU 10g impactor dropped from 12.5 mm (Gruner, 1992; Young, 2009). Behavioral function was then tested at weeks 1, 3, and 6 (although data from week 1 are unavailable; Ferguson et al., 2013).

Since our analysis here is illustrative only, we focused on one variable only; we selected hindlimb stride length because it is a widely-used outcome after spinal cord injury. We used the week 3 data as baseline, then calculated the boundary value that would need to be breached to indicate a change in stride length that was “meaningful” (i.e., exceeded that which might occur spontaneously because of physiological and analytical variation). We then compared the recorded value at week 6 for each rat with the previously calculated boundary value for improvement (in this example an increase in stride length) to determine in how many rats stride length was meaningfully increased. These comparisons were presented in tables.

### Statistics

For each outcome variable, the pooled data from all time points in all animals were evaluated for normality using histograms and q-q plots and then analyzed using standard methods to partition the interindividual and intraindividual variation (Fraser, 2001). In this type of investigation, the “analytical variation” relating to variation in equipment function cannot be estimated separately and so becomes included within the intraindividual variation. For most variables (those with a normal distribution), the raw data were entered into a mixed linear regression model with each animal entered as a random effect (Stata 14, StataCorp Ltd). The intraindividual coefficient of variation was derived as usual (i.e., SD/mean) and then used to derive the reference change interval (RCI), which defines the upper and lower boundaries within which sequential measurements of the same variable may spontaneously vary within an individual, by using the previously described (Harris and Yasaka, 1983) formula of:

RCI = baseline +/− (baseline  *  RCV),

where *RCV* (reference change value) = CV_i_ * 2^0.5^ * Z_p_, and *CV*_i_ is the intraindividual coefficient of variation; *Z_p_* is the *z* score selected to set the desired stringency of the interval and conventionally is set to consider a 5% false positive rate acceptable, which corresponds to a *z* score of 1.96. (Although very widely used in biomedicine, the 5% false positive rate is arbitrary and could be set more stringently by altering the *z* score in the formula; doing this will reduce proportion of individuals flagged as showing intervention effects.)

For those variables with a non-normal distribution, the lognormal method was used (Fokkema et al., 2006), in which the upper and lower boundaries are calculated separately.

For our illustrative example on use of the RCI, we compared stride length at week 3 and week 6 in the odc-sci.org SciCrunch database using a paired Student’s *t* test.

It is evident, and previously documented (Batka et al., 2014), that many commonly used CatWalk outcome variables may be correlated with each other (for instance, run duration and stride length), or with the time to cross the walkway, and so we determined the Pearson correlation coefficients for these interrelationships. We also wished to determine the variability in other, less commonly-used, methods of analyzing outcome after spinal cord injury that might be considered to provide evidence of the coordination between different limbs. Finally, we examined whether these other measures of coordination were correlated with run duration or run speed. Sample size decisions for calculation of RCIs are not well defined, partly because different variables have different ratios between analytical and within-individual variability (Røraas et al., 2012), but repeated measurements on relatively small numbers of individuals are known to provide satisfactory precision (Fraser and Harris, 1989; Braga and Panteghini, 2016). Specifically, it is recognized that increasing repeat testing on individuals is preferable to enrolling more individuals (Røraas et al., 2012). In this experiment, we analyzed three runs of 16 rats (therefore all were pair-housed) on each of eight occasions, following a period of training to competency.

## Results

We recorded data on three runs at each of eight weekly time points from all 16 rats included in this study, resulting in a pooled dataset of 384 measurements for each variable; the complete results are available online at odc-sci.org (doi:10.34945/F54S3W). In the data as a whole, there was evidence of considerable variability, as might be expected, and this can be summarized by describing means, ranges, etc. However, such analysis fails to take account of the auto-correlation between repeated measurements made on the same individual. The mixed model repeated measures analysis used in this experiment extracts this information and partitions variability into that within and that between individuals. The PubMed search using the terms listed above detected 57 hits; from these, the most commonly-used outcome measures were the following: base of support, stride length, regularity index, print area, duty cycle, swing duration, swing speed, maximum contact area, stance duration, and mean intensity; in addition, we examined run duration and average speed because of their relationship with many of these other variables. Each of these variables was then analyzed to derive a RCV.

For these commonly-reported outcomes (not including the regularity index), the RCV, the amount by which a normal individual might vary between repeated measurements, varied between 20% and 137% of baseline values (Table 3). Data from both hindlimbs were analyzed to assess repeatability, and, as would be expected, the RCVs were similar between limbs (Table 3). We could not assess the regularity index using this method because it is a percentage outcome with 100% being regarded as normal. The definition of 100% as normal implies a ceiling effect that creates an obstacle to quantifying variability.

**Table 3:** RCVs

Test	Mean	RCV (%)
Overall measures of hindlimbfunction		
Run duration	3.29 s	69.3
Average speed	36.87 cm/s	72.5
Base of support	2.71 cm	34.4
Coupling RHRF	45.12%	31.6
Coupling LHLF	45.40%	30.8
Hindlimb function, right		
Stride length	17.68 cm	29.1
Print area	1.82 cm^2^	65.0
Swing duration	0.16 s	25.7
Swing speed	112.52 cm/s	34.8
Stance duration	0.23^*^ s	Up: 121.5;down: 54.9
Max contact area	1.39 cm^2^	73.2
Mean intensity	103.61 AU	19.6
Duty cycle	58.60%	24.2
Hindlimb function, left		
Stride length	17.71 cm	27.1
Print area	1.83 cm^2^	66.1
Swing duration	0.16 s	27.2
Swing speed	112.45 cm/s	31.0
Stance duration	0.23^*^ s	Up: 136.6;down: 57.7
Max contact area	1.41 cm^2^	71.5
Mean intensity	103.63 AU	20.4
Duty cycle	58.33%	24.9

RHRF, right hind/right fore; LHLF, left hind/left fore; AU, arbitrary units.

^*^ indicates median value, not mean.

There was strong and significant correlation between most popular outcomes and the run duration, the exceptions were base of support and mean intensity (Table 4), both of which quantify aspects of paw placement. As expected, and previously reported (Batka et al., 2014), variables such as run duration, (limb) swing speed and stance time, were strongly correlated with run speed. Most of the popular outcome measures were closely intercorrelated. Important exceptions were the poor correlations between base of support and print area with swing duration and that between most measures of limb motion (except stride length) and mean intensity.

**Table 4:** Pearson correlation matrix for commonly measured variables, RH

	Runduration	Stridelength	Base ofsupport	Printarea	Swingduration	Swingspeed	Maxcontact	Stancetime	Runspeed	Meanintensity	Dutycycle
Run duration	**1**										
Stride length	**–0.454**	**1**									
Base of support	0.090	**–0.268**	**1**								
Print area	**0.219**	**–0.140**	0.098	**1**							
Swing duration	**0.218**	0.0207	0.046	–0.004	**1**						
Swing speed	**–0.487**	**0.720**	**–0.223**	–0.071	**–0.660**	**1**					
Max contact	**0.183**	**–0.107**	0.062	**0.97**	–0.021	–0.039	**1**				
Stance time	**0.568**	**–0.558**	**0.260**	**0.202**	**0.202**	**–0.546**	**0.354**	**1**			
Run speed	**–0.770**	**0.588**	**–0.161**	**–0.326**	**–0.326**	**0.660**	**–0.305**	**–0.716**	**1**		
Mean intensity	0.057	**0.123**	**0.115**	**0.509**	0.016	0.090	**0.579**	0.079	–0.060	**1**	
Duty cycle	**0.437**	**–0.673**	**0.235**	**0.515**	**–0.176**	**–0.361**	**0.458**	**0.773**	**–0.617**	**0.114**	**1**

Bold indicates *p* < 0.05.

**Figure 1.**
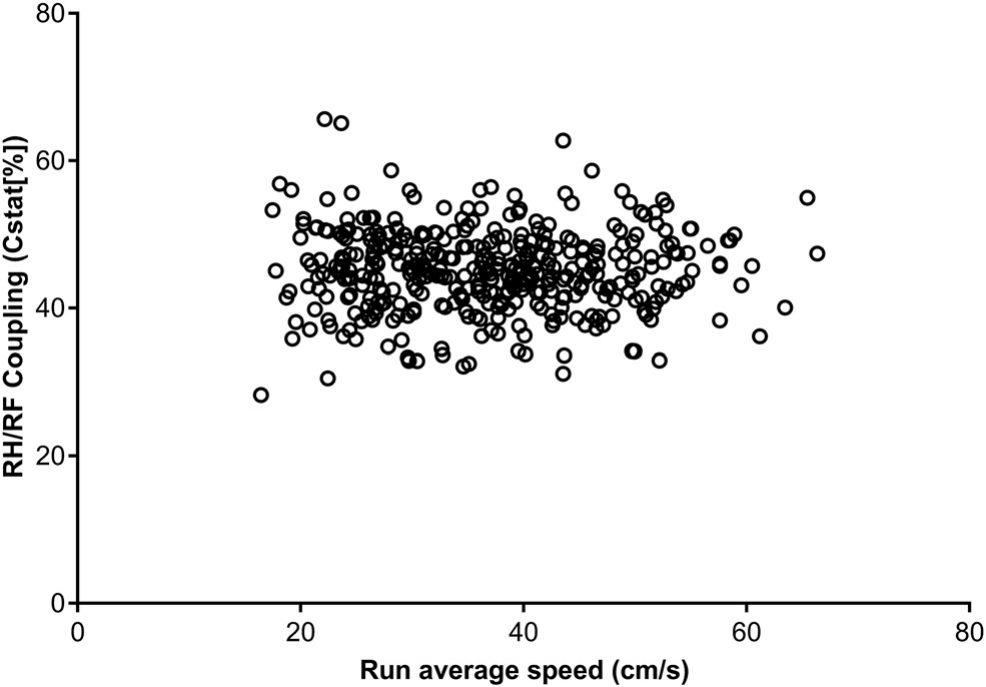
Scatter plot between run speed and right hind/right fore (RH/RF) coupling in normal rats on the CatWalk. There is no apparent correlation between these variables (*r* = −0.012; *p* = 0.885).

Kinematic data can be used to examine the strength of temporal relationships between movements in different pairs of limbs (Diogo et al., 2019), and there are similar data available from CatWalk that might be helpful in analyzing outcome following thoracolumbar spinal cord injury. In particular, CatWalk produces many measures of the temporal relationship between placement of two specific paws (see Batka et al., 2014), and which can be expressed as a percentage of contact time of one paw during the step cycle period of another. Some of these relationships are summarized as circular statistics (e.g., “CStat mean”; Fig. 1) and can take values between 0 and 100. As an example, we determined that coupling between right hindlimb (RH) and right forelimb (RF) had a similar RCV to other popular variables: 31%. There was no apparent correlation between run speed and RH-RF coupling interval (*r* = −0.012; *p* = 0.885; Fig. 1).

### Illustrative example

In order to provide a more concrete example of the use of individual analysis, we applied our results to open source data provided on the odc-sci.org SciCrunch database (https://scicrunch.org/odc-sci/lab/view-dataset?labid=51&datasetid=131). These data are derived from rats that had unilateral C5 level spinal cord injuries and were then tested on the Catwalk at weeks 3 and 6 after injury (week 1 data were not available for logistic reasons during the original experiment). Rats in this database did not receive any test intervention. In the specific example we show below, the data are those for RH stride length following NYU impactor injury (Gruner, 1992; Young, 2009) with a weight drop of 12.5 mm.

**Figure 2.**
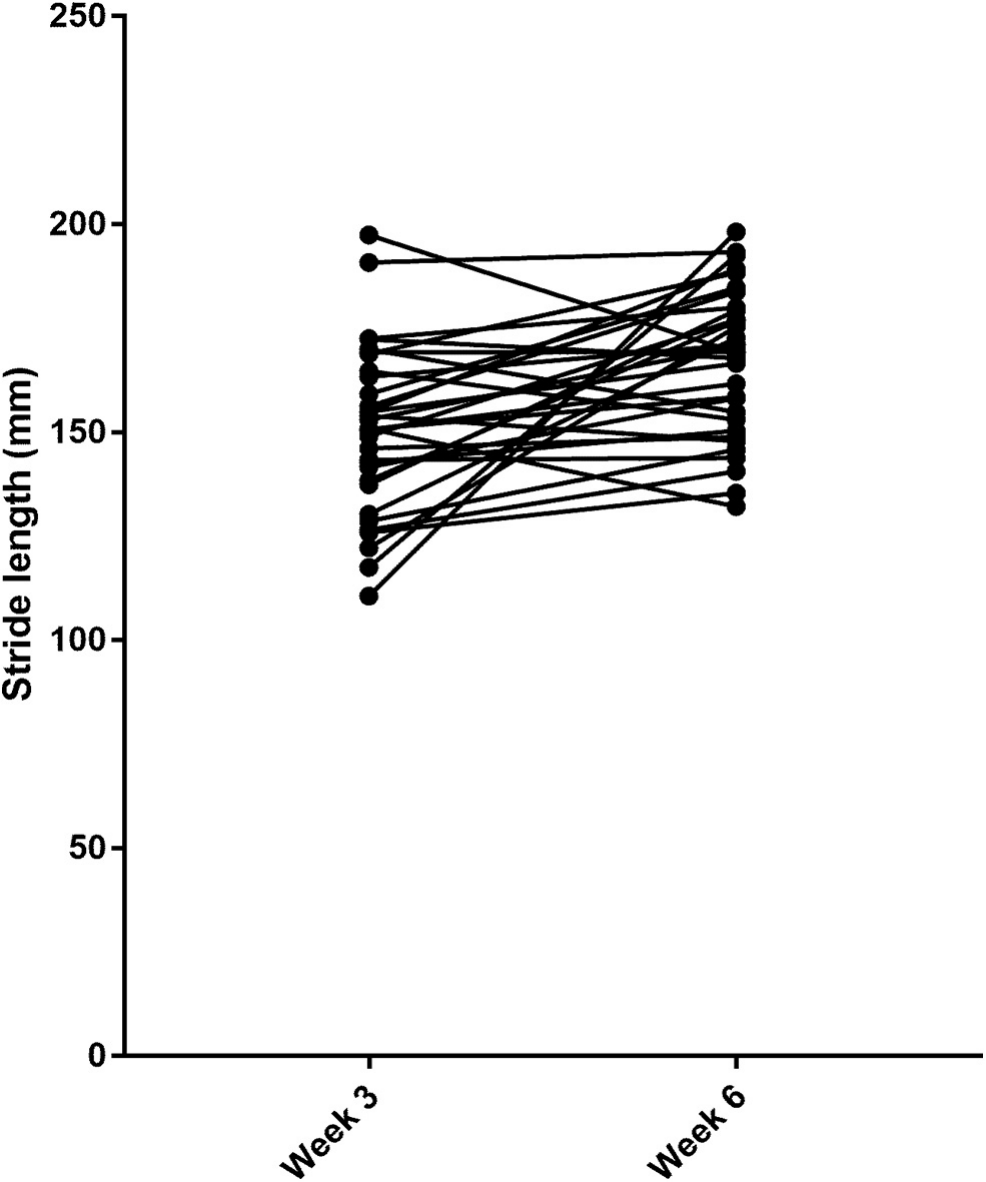
RH stride length at week 3 and week 6 after rats had received a unilateral C5 spinal cord impact injury (SciCrunch data).

The analysis of our normal rats defined that, for animals at a functional plateau, the RCV for hindlimb stride length is 28%, implying that a change of 28% or more from baseline value is necessary to indicate a meaningful change. As can be seen in Table 5, this difference is attained by five of 32 rats within the tested group. Conventional analysis by paired sample Student’s *t* test shows that there is a significant difference (means: week 3, 150.4 mm; week 6, 166.8 mm; *p* = 0.0009) between the two time points (Fig. 2). A meaningful change (i.e., more than would be expected from analytical and physiological fluctuations alone) in 16% (5/32) of animals is more than would be expected by chance [the RCI boundaries are set with a 95% confidence interval (two tails of *z* score of 1.96) implying that, on average, values for only 2.5% of the population would exceed the upper boundary]. Nevertheless, the change in function between week 3 and week 6 is not meaningful for 84% of animals, consistent with the majority of rats reaching a functional plateau on this outcome measure between three and six weeks after injury.

**Table 5:** Application of RCI analysis to previously published data on RH stride length following unilateral 12.5-mm NYU impactor injury at C5

Ratnumber	Week 3 Stridelength (mm)	Week 6 Stridelength (mm)	RCV (fromour study)	Upper RCIboundary(= week 3 + RCV)	Lower RCIboundary(= week 3 – RCV)	Week 6 exceedsupper RCIboundary?	Week 6 lessthan lower RCIboundary?
1	150.70	158.39	42.20	192.90	108.50	No	No
2	159.17	184.74	44.57	203.74	114.60	No	No
3	138.41	176.61	38.76	177.17	99.66	No	No
4	150.63	161.65	42.18	192.81	108.46	No	No
5	146.08	148.88	40.90	186.98	105.18	No	No
6	143.36	143.85	40.14	183.50	103.22	No	No
7	169.21	169.29	47.38	216.58	121.83	No	No
8	168.78	188.33	47.26	216.04	121.52	No	No
9	169.94	154.81	47.58	217.52	122.36	No	No
10	197.48	169.24	55.29	252.77	142.19	No	No
11	190.84	193.31	53.43	244.27	137.40	No	No
12	128.59	145.83	36.00	164.59	92.58	No	No
13	172.51	180.00	48.30	220.81	124.21	No	No
14	137.35	**179.32**	38.46	**175.80**	98.89	Yes	No
15	122.18	**175.32**	34.21	**156.39**	87.97	Yes	No
16	110.61	**198.19**	30.97	**141.58**	79.64	Yes	No
17	117.51	**192.55**	32.90	**150.41**	84.61	Yes	No
18	125.85	135.39	35.24	161.09	90.61	No	No
19	142.68	150.32	39.95	182.63	102.73	No	No
20	153.95	147.86	43.11	197.06	110.85	No	No
21	153.02	170.64	42.85	195.87	110.18	No	No
22	154.96	166.54	43.39	198.34	111.57	No	No
23	154.82	189.25	43.35	198.18	111.47	No	No
24	149.06	176.97	41.74	190.79	107.32	No	No
25	126.54	140.62	35.43	161.97	91.11	No	No
26	156.21	183.76	43.74	199.95	112.47	No	No
27	163.30	170.99	45.72	209.02	117.57	No	No
28	130.30	**172.69**	36.49	**166.79**	93.82	Yes	No
29	150.85	132.10	42.24	193.09	108.61	No	No
30	164.72	153.03	46.12	210.85	118.60	No	No
31	172.34	167.85	48.26	220.60	124.09	No	No
32	141.57	158.13	39.64	181.21	101.93	No	No

In this example, change in function was generated by time alone, but the same principle could be used in other experiments to determine the proportion of individuals that exceed boundary levels of function following an intervention.

## Discussion

This analysis of widely-used CatWalk outcome measures can enrich interpretation of experiments through provision of additional viewpoints on the data, therefore increasing robustness of analysis. In this experiment, we defined boundary limits of spontaneous variability in outcome measures within individual animals as they complete the CatWalk test. These boundary limits can then be applied, as we demonstrate in our example, to determine how many animals within an experimental group achieve meaningful change from baseline function and provides context to interpret the magnitude of that change. The ability to define outcomes in specific individuals and to define the proportion of individuals that have exceptional outcomes that is provided by this method complements standard analysis of group-level outcomes. Using the same dataset an investigator acquires two lines of evidence regarding intervention effect: the overall group effect and the proportion of individuals that show exceptionally good (or bad) outcomes.

First, the large RCIs associated with many of the investigated CatWalk outcome measurements implies that only substantial changes from baseline would provide evidence for an intervention effect in any specific test individual. As we show in our illustrative example, this interpretation may, at first sight, seem at odds with the interpretation derived from routine examination of group-level data. The explanation of this difference is that, while there may be an improvement in measured function in many subjects in a group that is associated with a significant change on a standard statistical test, in contrast, at an individual level each subject may improve by less than that which occurs spontaneously as natural variability in function. This is not to say that the group-level difference should be ignored, just that the individual-level analysis provides additional information; in our example, for instance, it demonstrates that only a small proportion of the subjects make improvements beyond that which might be anticipated because of stochastic behavioral variation. The realization that only substantial changes in individual function are meaningful for many of these outcomes also aids in interpreting the magnitude of effect observed throughout the group as a whole. For instance, the group effect we detected in the illustrative example was a change in mean stride length of ∼15 mm, which amounts to ∼10% of the baseline (week 3) stride length. Comparison with the RCV of 28% implies that the detected group level change is small when viewed in the context of the variability of an individual’s limb function.

RCI analysis of this type may be helpful for many experiments that are designed with an eye on translation to the clinic. To be therapeutically successful, clinical interventions (most relevantly here for spinal cord injury) need to have a noticeable benefit on individual patients (although this might also depend on cost-benefit ratio; Steeves et al., 2012). For instance, a patient who is asked to consider receiving an intraspinal allograft cell transplant (that would carry considerable potential risk) would be likely to want to receive greater functional improvement than might be the current difference between their disability on a “good” versus a “bad” day. Therefore, this individual-level analysis can aid in increasing the rigor with which putative therapeutic interventions are selected to go forward to clinical trials. Use of CatWalk outcome measures in this context might be questioned, because only rats that have reasonable ability to walk can complete the CatWalk test, and, as such, these animals may not appropriately model severe spinal cord injury in humans. For that reason, intervention benefit detected by CatWalk might not imply similar benefits would accrue in severely spinal cord-injured individuals (including people). On the other hand, analysis using the RCI as described here can provide greater confidence in intervention effect and such reliable identification of an effect in any incomplete injury could be used as a first step to suggest similar benefit in incompletely injured humans.

A second major benefit of using the individual-level analysis is to aid in designing efficient experiments, through two main routes. First, in the example dataset, we can identify specific rats in which there was a meaningful change in stride length between week 3 and week 6. Examining the data suggests that those individuals had relatively short stride lengths at week 3, and this information could be used to make future experiments more efficient. So, if spontaneous increase in stride length was largest in those with short strides at week 3, it would be advantageous to exclude such animals if the test intervention was thought likely to increase stride length: the individuals most likely to show spontaneous improvement will only add noise to the expected intervention signal. An alternative explanation might be that there is a ceiling effect in this dataset, such that many animals have already attained a “normal,” or near-normal, stride length by week 3 after injury and that there is little scope for improvement by week 6. If this were the case, which could be confirmed by testing animals at later time points, then it would suggest that the experiment would be more efficient if a more severe injury model was used.

We are aware that our analysis of the illustrative example assumes that we can apply the RCIs derived in our laboratory to data derived elsewhere and stress that we are simply using it as an example. Ideally, all laboratories would derive their own RCIs, because the precise conditions in which rats are tested may vary and so measurement variability within and between individuals might also consequently vary. However, this might not always be practical and an alternative approach is for training and testing methods to be standardized as much as possible between laboratories to facilitate comparison. Even so, there are many reasons to consider that RCIs are largely an inherent property of the parameters that are measured, a well-recognized feature in clinical medicine (Ricós et al., 2004), and are relatively robust. First, the RCI is derived from coefficient of variation, which standardizes variation against the mean within the same dataset, meaning that small changes in mean values will have little effect. Second, variability in sick individuals at a plateau is recognized to be generally similar to that in healthy individuals (Fraser and Harris, 1989), and, in human medicine, it is not generally necessary to construct individual RCIs for different groups of people (e.g., by age, ethnicity, etc.) because they are associated with minimal effects (Jones, 2019). It is recognized that in acute sickness, some measured values are more variable than they are in health (Ricós et al., 2017), but the effects on decision-making would be to make this individual-level analysis more (rather than less) sensitive than it should be (i.e., it will falsely identify too many individuals as exceptional). Finally, as others have noted (Ricós et al., 2004), a breached reference change boundary should be interpreted in combination with other factors, such as, in this context, group-level analysis, rather than as a brightline delineation between “abnormal” and “normal.”

When considering the future implications of our analysis of CatWalk data, an “ideal” outcome measure would unequivocally quantify an aspect of spinal cord function and have a high level of precision and low intraanimal and interanimal variation, meaning that any changes in function induced by an intervention would be easily detected. Furthermore, if a battery of tests is to be used, it is important that each item should be independent. In this experiment we examined many of the most popular CatWalk outcomes and few meet all these criteria. First, many of these measures have high intraanimal variability, many have RCVs >50%, indicating a need for substantial change from baseline to define an effect greater than could be attributed to spontaneous variation. Those outcome measures with high RCVs are likely to prove insensitive to intervention effects. It is noteworthy that the variability in many outcomes was large despite us setting reasonably stringent rules about “compliant” walkway traverses.

Another difficulty is that many of the most popular CatWalk outcomes are correlated with each other, presumably through a mutual dependence on run duration or run speed. Although this is not necessarily a problem if just one of these variables is used alone, it does become more problematic if several are used in a battery of tests since, essentially, they are all providing similar information. On the other hand, we have found that some of the kinematic-like measures, such as the coupling between specific pairs of limbs, have reasonably low RCVs and so might be relatively sensitive in detecting effects of lesions of interventions. Furthermore, measures of limb coupling across the lesion site (i.e., fore and hind coupling) have the advantage that they are likely to measure aspects of spinal cord function that are susceptible to disruption by a thoracic lesion (Diogo et al., 2019). As we demonstrate here, they also have the merit of not being susceptible to changes in run duration/run speed.

An important aspect of designing experiments is having predefined outcome measures, as would be standard practice in clinical trials (Kendall, 2003), although in laboratory studies, it is also necessary to consider the balance between exploratory and confirmatory intent (Kimmelman et al., 2014). CatWalk offers a plethora of variables to choose from, and if outcome measures are not predefined, there is the risk that detected positive results might reflect random effects selected by the researcher after data generation (Wicherts et al., 2016). For this reason, it is essential for CatWalk experiments that the variables that will be used to determine the efficacy of an intervention are defined before the study commences and, also, if possible, the magnitude of change that can be defined as meaningful is also predefined. Based on our analysis presented here, it would seem prudent to select outcomes that have minimal intraanimal variability and also not to restrict analysis only to outcomes that are inevitably correlated by their dependence on run speed (or duration).

Therefore, based on our results, we would suggest using stride length or swing duration and base of support or duty cycle as appropriate measures of hindlimb use following thoracic spinal cord injury, plus using hindlimb-forelimb coupling as a kinematic outcome that might be expected to quantify coordination mediated by the injured region of the spinal cord. The results we present here might also be helpful for defining minimum difference between groups in sample size calculations for future experiments using these outcome variables.

Finally, as a limitation to this form of analysis, it is important to note that the derivation of RCIs is dependent on calculation of the within-individual coefficient of variation that, in turn, depends on calculation of standard deviation. This implies a need for continuous numerical data and a range of values in normal individuals that does not include a floor or ceiling. Thus, commonly-used behavioral outcomes used in spinal cord or brain injury models that quantify times, distances, angles, or forces, such as the rotarod, water mazes, open field maze, joint or limb position or kinematics, grip strength, and sticky label removal, are clearly amenable to this analysis of variability. Non-behavioral tests such as electrophysiological measures and quantification of components of body fluids can also be analyzed by this method, although there is a requirement for repeated measures on normal animals, which must not in themselves be a cause of variation (e.g., repeated CSF sampling). Count data are less amenable, because outcomes are integers, but they can often be easily converted into counts per unit time or distance, and so the method may be adapted for the forepaw reaching, cylinder (rearing) and beam walking tests. It is also important to highlight that, although it is most straightforward to derive RCVs from normally distributed data, the method can be applied to non-normal data by using the log-normal method (Fokkema et al., 2006).

However, for two reasons, analysis of individual variability by calculation of a RCV is not appropriate for outcomes that are derived from a scoring scale, such as the “BBB scale” (Basso et al., 1995), the (modified) neurologic severity scale or the Bederson scale (Bederson et al., 1986). First, by definition, normal animals almost invariably score at the floor or ceiling of these scales meaning that it is not possible to determine “expected” variability and, second, the attributed scores are not truly numeric and so the standard deviation has an uncertain meaning. Instead, for this type of outcome measure, population-based reference intervals can be used to define boundaries within which defined proportions of the outcome values will fall at specific times after specific injuries (Jeffery et al., 2020), although such methods require much larger sample cohorts.

## Synthesis

Reviewing Editor: Deanna Smith, University of South Carolina Decisions are customarily a result of the Reviewing Editor and the peer reviewers coming together and discussing their recommendations until a consensus is reached. When revisions are invited, a fact-based synthesis statement explaining their decision and outlining what is needed to prepare a revision will be listed below. The following reviewer(s) agreed to reveal their identity: David Magnuson, Mark Ballermann.

The reviewers agree this is a well written paper and are enthusiastic about the potential usefulness of the proposed method of analyzing behavioral data to identify how variability correlates with post therapy improvements in spinal cord injury research. The concept that variability in the control group (or pre-injury group) can drive the strength of the post-injury analysis is important. The reviewers conclude that the manuscript is of sufficient quality to be published in eNeuro with some changes following rereview. The concerns can likely be addressed without further experimentation by more fully exploring the advantages and limitations of the system. For example, the approach may be too narrow with respect to types of injuries and outcome measures that are practical, and it may be difficult for labs to find appropriate baseline databases. Also, the approach may not be applicable to non-kinematic measurements or solve the problem of high variability. The authors should show the actual results for swing duration, base of support and duty cycle, and the selection of a defined z-score designed to set a false positive rate at 5% may rest on assumptions that are not fully understood. This should be addressed in the discussion. The full critiques by each reviewer are included below.

Reviewer 1:

This is a well written paper addressing an important and potentially meaningful set of issues for SCI pre-clinical and translational research. The ability to detect meaningful improvement at the individual level as well as from between group comparisons is a critical need in SCI research, both human and animal. This approach would be of great benefit and an ideal addition in the area of human SCI recovery due to the heterogeneous nature of this group. The authors propose the use of a reference change value to compare to an individual score in order to show improvement which would offset the drawbacks of our most common approach at present that relies heavily on significant group differences to show improvement. Its use in conjunction with standard between groups comparisons not only offers another useful measure to determine significant improvement but enables examination at the individual level as well, an approach that is, in general, not widely embraced.

The primary weakness of the work is the appearance that the proposed approach may only be appropriate to a relatively narrow group of experiments (mild contused injuries) using a relatively narrow group of outcome measures that may not be available in all labs. The focus on mild injuries is fine as an example, but limits the translational strength of these efforts, this exposing a disconnect with the premise provided in the introduction.

Potential drawbacks

-If a laboratory does not have an extensive database from which to draw baseline values, they would need to find access to other databases, as used in the present work. This approach is feasible as long as the database utilized includes baseline data that coincides with the needs of the laboratory’;s research goals (i.e., age, gender, strain, level, type and severity of injury, etc).

-The calculation for each subject to determine if the amount of change is greater than the amount of change at baseline entails considerable work, depending on the number of measures and the number of timepoints during recovery being compared. Although the authors suggest limiting the number of outcome measures to those that are independent of one another, by and large, greater information is gained by increasing the number of outcome measures. Although many measures do overlap in the information attained, this is precisely where specific detailed knowledge is gained regarding aspects of recovery. For example, in kinematic coupling measures, forelimb to hindlimb as well as hindlimb to forelimb step patterns for coordination (diagonal), sidedness (right or left side) and forelimb and hindlimb alternation all add valuable information to help explain details of the complexities occurring during recovery. Moreover, valuable information can be gleaned from correlative measures. When correlated measures are observed in an individual with improvement, these results can be compared to other individuals showing improvement but do not show the same correlation between the same outcome measures.

-The authors suggest 3 other low variability measures for use with this procedure besides stride length: swing duration, base of support and duty cycle. The paper would be greatly enhanced if results for these other outcomes were shown also in the paper for comparison, at the very least, in a table. Were these overall means also significantly different? Did they also reveal low proportions as seen for stride length (5/32)?

-How applicable is this approach to other types of outcome measures? Many laboratories do not use kinematics due to its expense and extensive time requirements for video analysis. It would be informative to see a different type of outcome measure applied to this procedure for comparison.

-High variability is always a hindrance to achieving significant differences in between groups analysis. However, that problem is not solved here since measures with low variability must be used with this procedure.

Minor comments

-pg. 9, line 202-The use of the term “paired Student’;s t-test” is incorrect. The proper term for independent t-test comparisons is either “Independent t-test” or “Student’;s t-test” because the Student’;s t-test is applicable only for comparisons between independent means, whereas paired t-tests are only applicable for matched mean pairs.

-pg. 9, line 217- It is stated that it is preferable to use repeated measures compared to enrolling more subjects for independent comparisons, but the disadvantages are that the results are less generalizable to the population. Please comment/expand on this.

-pg. 10, line 233- It is unclear exactly what terms were used in the PubMed search, please specify.

-pg. 12, line 291- I believe the idea of including comparisons of the proportion of individuals with improvement is a very important point which is too often overlooked, and this platform offers the opportunity to emphasize the point more strongly to the SCI research community. Though inherently nonparametric, binomial proportion tests between 2 independent proportions is an ideal way to incorporate and further show the utility of this procedure where mean comparisons are inadequate. I would strongly endorse expansion of this idea to highlight the proportions of subjects with improvement. [For example, see potential drawbacks section above, point 3, where this could be done for the other 3 outcome measures]

-pg. 13, line 321- Here it is suggested that in light of a baseline change of >28% (reference change value), that a measure with only a 10% change “may not be very important in terms of overall limb function.” I would submit that very small percentages of change can be very important and relevant but is dependent upon the specific outcome measure. For example, changes in the BBB scale of only 2 points can be very relevant to improvement in the intermediate stage of recovery (e.g., values between 8 and 10 distinguish the emergence of weight support and stepping ability). Please comment/expand on this.

-pg.,13, line 327- The first benefit for this procedure mentioned is with respect to translational use to SCI patients. The data in the present paper utilizes mild-moderate injury (12.5g/cm contusion). The SCI patient population is interested primarily in the change from non-weight support to weight supported stepping, a more moderate injury paradigm. There is a bit of a disconnect here in relevancy (2 very different injury severities) that is not really addressed. Are the suggested approaches appropriate only to milder injuries using the CatWalk or could it be applied to other measures appropriate for more severe injuries. Please comment/expand on this.

Reviewer 2:

The authors spend a good deal of space picking apart the concepts of clinically significant and statistically detectible changes in measuring of rat gait using the Catwalk tool. They use the concept of Reference Change Values and Intervals collected in their own laboratory in conjunction with previously published data on spontaneous recovery of hindlimb function in rats to determine that the threshold of ‘;meaningful recovery’; was met in 5 of 32 rats. It would appear that the reader is left to decide the applicability of these results. In particular the recommendations of stride length, duty cycle, and swing time are noted as being strongly correlated with walking speed. Forelimb / hindlimb coordination is also recommended at the end of the discussion as an important measure which is plausibly related to recovery in thoracic models of spinal injury.

My general sense is that this is a worthwhile contribution and is of sufficient quality to be published in eNeuro with a number of changes defined below. An issue I think may be problematic with the premise of the approach is that the selection of a defined z-score which is designed to set a false positive rate at 5% may rest on assumptions that are not fully understood. This should be addressed in the discussion.

Minor

Abstract

I am unsure if the reference to ‘;real change’; on line 17 has a scientific meaning. Perhaps rewording to state something like recovery that meets a predetermined criteria. It may be most helpful for readers to state this in the abstract if possible.

Significance statement

The second sentence inappropriately uses ‘;While’; to mean ‘;Although’;.

Introduction

The final paragraph of the introduction seems quite unclear in terms of what the researchers in fact completed in the present work. As is, the sentences reference general principles of statistical analysis, and the philosophy of comparing individual measures to group which had been adequately introduced at that point. I gather that a publication survey was completed, and the results from the survey was used to define bounds for variability in performance when measured in the Catwalk test by intact, young adult, male Sprague-Dawley rats. This last bit was not in the introduction, and I think should be to help the reader assess generalizability and therefore usefulness of the findings.

Methods

The method for calculating forelimb / hindlimb coordination should probably be defined somewhere in the methods as it forms the basis for Figure 1. I am unfamiliar with CStat as an outcome value.

The light-dark cycle (e.g., usually something like 12 hr light 12 hr dark with the light cycle beginning at 0700) should be stated in the Subjects section.

This sentence does not make sense (lines 147-149). ‘;We collected .... data .... by searching Pubmed...’; I think it should probably state something like the outcome measures were based on those previously published which had been located using a PubMed search.

Line 214 has a typo - ‘;measurands’;.

Tables 1 and 2 seems to have information that would normally be included in the manual for the apparatus and probably unnecessary. One important exception is the duration criteria for runs, however.

Results

Table 3 presents the reference change values absent the group means used to calculate them. It might be fairly straightforward to provide these values so as to support the reader in interpreting the novel data. The wide time window from 0.5 seconds to 5 seconds may permit varying gaits to be employed by the rats, and these results may help the reader understand the extent to which they would want to apply this analysis in their own experimental setting.

Discussion

It’;s unclear what exactly is meant when the authors state that ‘;additional viewpoints on the data .... increasing robustness of the analysis’;.

The final few sentences appear to form the conclusion of the manuscript, which may be most suitable standing as a separate paragraph.
